# Ex vivo and computational investigation of corneal iontophoresis to enhance penetration of high-molecular-weight compounds: a study using albumin as a model molecule

**DOI:** 10.1038/s41598-026-43580-y

**Published:** 2026-03-31

**Authors:** Asmaa K. Mohamed, Sherif S. Mahmoud, Shaimaa M. Elshibly, Gehan M. Kamal

**Affiliations:** 1 Physics department, Faculty of Science, Al-Azhar University (Girls Branch), Cairo, Egypt; 2 Biophysics and Laser Science Unit, Research Institute of Ophthalmology, Giza, Egypt

**Keywords:** Cornea, Iontophoresis, Albumin, FTIR spectroscopy, Temperature modeling, Macromolecular delivery, Thermal safety, Biophysics, Materials science, Medical research

## Abstract

Efficient ocular drug delivery remains a major challenge due to the strong barrier properties of the corneal epithelium and the rapid clearance of topically applied formulations. This limitation is particularly critical for hydrophilic and high-molecular-weight (HMW) therapeutics such as proteins and biologics. In this study, we investigated transcorneal iontophoresis as a strategy to enhance the delivery of HMW compounds using albumin (66 kDa) as a model macromolecule. Ex vivo rabbit corneas were exposed to iontophoretic currents ranging from 0.5 to 7 mA for clinically relevant conditions, while an extreme non-clinical current (500 mA) was included as a destructive positive control to model irreversible tissue disruption. Albumin permeation was quantified by intrinsic fluorescence spectroscopy, and corneal structural responses were assessed using Fourier-transform infrared (FTIR) spectroscopy. In parallel, computational thermal modeling based on the Pennes bioheat equation was employed to evaluate current-dependent Joule heating and thermal safety margins. Results demonstrated that iontophoresis significantly enhanced albumin transport compared with passive diffusion, with increased permeation at higher currents, particularly at 6–7 mA. Thermal simulations indicated that currents ≤ 2 mA maintained corneal surface temperature within the normal physiological range (32–36 °C), while currents ≥ 3 mA progressively increased corneal temperature, approaching established thermal stress thresholds depending on exposure time. FTIR analysis revealed current-dependent spectral alterations consistent with changes in hydration dynamics, protein conformational environment, and lipid organization, suggesting reversible molecular adaptation at low-to-moderate currents and pronounced disruption at extreme electrical exposure. Collectively, this study identifies a practical current-time operational window for transcorneal iontophoresis that enhances macromolecular transport while maintaining corneal thermal and molecular integrity, supporting its potential for non-invasive ocular delivery of biologic therapeutics.

**Supplementary Information:**

The online version contains supplementary material available at 10.1038/s41598-026-43580-y.

## Introduction

Efficient ocular drug delivery remains a major challenge in ophthalmic pharmacotherapy due to the unique anatomical and physiological barriers of the eye. The corneal epithelium, tear turnover, conjunctival clearance, and blood-ocular barriers collectively limit the penetration of topically applied drugs into intraocular tissues, resulting in poor bioavailability; <5%^1,2^. Conventional eye drops often fail to achieve therapeutic drug concentrations in the anterior and posterior segments, particularly for hydrophilic and high-molecular-weight (HMW) compounds^3^. Therefore, the development of non-invasive yet effective strategies to enhance ocular drug penetration is of significant pharmacological and clinical importance.

Despite major advances in ocular therapeutics, current topical formulations often fail to achieve therapeutic intraocular concentrations, especially for large hydrophilic molecules, resulting in reliance on invasive approaches such as intracameral or intravitreal injection. Such invasive delivery routes are associated with patient discomfort, procedural complications, and increased healthcare burden. Therefore, non-invasive enhancement technologies that enable controlled and safe penetration of high-molecular-weight therapeutics are increasingly needed for anterior segment diseases, including infectious keratitis, inflammatory corneal disorders, postoperative complications, and immune-mediated ocular surface diseases.

Ocular iontophoresis, initially introduced by Wirtz^4^, offers a promising method for transocular delivery of small and large molecules. It involves the application of a low-intensity electrical current (0.5–5 mA) to drive charged drug molecules through biological membranes via electrorepulsion (Nernst-Planck effect) and electro-osmosis, thereby enhancing transcorneal and transscleral permeability^5,6^. The technique can be applied transcorneally for anterior segment delivery or transsclerally for posterior segment targeting, depending on the site of electrode placement^7^.

The mechanism of ocular iontophoresis relies primarily on the interaction between the applied electric field and the physicochemical properties of both the drug and the ocular tissue. In addition to direct electrophoretic movement, iontophoresis induces electro-osmotic solvent flow that can assist the transport of neutral or weakly charged molecules^5^. This dual mechanism allows for a substantial increase in flux compared with passive diffusion, without the need for chemical enhancers or tissue disruption.

Several preclinical and clinical studies have demonstrated the safety and efficacy of iontophoresis for ocular drug delivery. For example, transcorneal iontophoresis of dexamethasone phosphate achieved therapeutic anterior chamber concentrations comparable to intracameral injections, while minimizing systemic exposure^8^. Similarly, transscleral iontophoresis of ciprofloxacin and vancomycin produced effective levels in posterior ocular tissues, suggesting its utility in the management of endophthalmitis and posterior uveitis^6,7^. Recent investigations revealed that controlled electrical application can transiently increase corneal and scleral permeability without causing significant tissue damage^9,10^.

The advantages of ocular iontophoresis are multifold: it allows precise control over dosing by adjusting current intensity and duration; it minimizes systemic absorption; and it provides a rapid, localized delivery route that can be repeated safely^5^. Furthermore, since the technique is compatible with both hydrophilic and HMW molecules, it offers an attractive platform for the non-invasive administration of emerging biologics and gene-based therapeutics^10^. Nonetheless, challenges remain in optimizing electrode design, ensuring reproducibility across species, and preventing current-induced irritation or corneal surface changes. Despite these advantages, challenges remain regarding optimal current density, formulation compatibility, and tissue tolerance during repeated treatments. Furthermore, the application of iontophoresis to HMW compounds such as peptides, proteins, or nanoparticles is still under investigation.

Although ocular iontophoresis has been explored for decades, most studies have focused primarily on drug concentration outcomes, with limited integration of thermal safety evaluation and molecular-level assessment of tissue integrity. Thermal safety is particularly important because Joule heating may cause protein denaturation, epithelial barrier breakdown, and irreversible corneal injury if current intensity and exposure duration are not properly optimized. Furthermore, structural investigations are often performed using histology, while spectroscopic approaches capable of detecting subtle hydration and protein-lipid changes are less frequently incorporated. Therefore, the current literature lacks integrated experimental-computational evidence defining a safe current-time window for macromolecular iontophoresis with simultaneous confirmation of molecular structural stability.

Accordingly, the aim of this study was to investigate the current-dependent enhancement of transcorneal delivery of a high-molecular-weight protein using albumin (66 kDa) as a model compound. This work integrates (i) ex vivo iontophoretic permeation experiments, (ii) computational thermal modeling using the Pennes bioheat framework to estimate Joule heating, and (iii) FTIR spectroscopy to assess current-induced molecular alterations in corneal tissue. The novelty of this work lies in defining a mechanistically supported operational window for transcorneal iontophoresis that balances enhanced macromolecular delivery with preservation of corneal thermal safety and structural integrity.

## Materials and methods

### Computational modeling of temperature distribution during corneal iontophoresis

Simulations were implemented in Python programming language, and the steady-state model incorporates coupled electrical-thermal phenomena in a multi-layered corneal structure, accounting for tissue-specific electrical properties, Joule heating, and heat transfer mechanisms. The cornea was modeled as a curved multi-layered structure consisting of three distinct tissue layers and an overlying tear film. This is justified by the large radius of curvature (0.6 mm) relative to the total thickness (0.5 mm). The layer dimensions and arrangement are summarized in Table 1.

**Table 1:** Corneal layer dimensions and arrangement.

Layer	Thickness (µm)	Position from anterior
Tear film	12	0–12
Epithelium	50	12–62
Stroma	430	62–492
Endothelium	20	492–512

The current density *J* through the corneal layers was calculated according to Eq. (1), where *I* is the applied current, and *A* is the cross-sectional area of the cornea. Moreover, the electrical resistance *R* of each layer was determined using Eq. (2). where *L* is layer thickness and *σ* is electrical conductivity.

1 $$J=I/\mathrm{A}$$

2 $$R_{{layer}} = L/\left( {\sigma \times A} \right)$$

The electrical properties involved in the calculations are given in Table 2.

**Table 2:** Electrical properties of corneal layers.

Layer	Electrical conductivity (S/m)	References
Tear film	1.5	^11–13^
Epithelium	0.0004	^12,14,15^
Stroma	0.25	^14,16,17^
Endothelium	0.0066	^17,18^

The voltage drops *ΔV* across each layer was calculated using Ohm’s law: $\Delta V = I \times R_{{layer}}$. The temperature distribution was modeled using the Pennes bioheat equation in one-dimension. Details are provided in the Supplementary Materials. The one-dimensional Pennes bioheat equation for the cornea, considering radial heat flow, is a partial differential equation that describes temperature distribution *T* over time *t* and position *r*^19,20^. Temperature distribution was simulated for current intensities of 0.5, 1, 2, 3, 4, 5, 6, 7, and 500 mA at time intervals of 10, 30, and 60 s. The simulation runs for 60 s of physical time using 0.1-second time steps, requiring 600 computational iterations that take 2–3 s to complete. Both convective and evaporative cooling (tear film/air interface) and perfusion cooling to body temperature (endothelium/body interface) were considered in the simulations.

### Ex vivo corneal iontophoresis

Chinchilla rabbits (2–2.5 kg, both genders) were obtained from Al-Azhar University (Egypt) and enrolled directly in the experimental procedure. The experimental protocol received ethical approval from the research ethics committee of faculty of Medicine for girls at Al-Azhar university (Study ID: 3002, with approval code RHDIRB2018122001), following ARVO guidelines for the use of animals in ophthalmic research, and the study is reported in accordance with ARRIVE guidelines. Animals were randomly divided into ten groups, each with 5 rabbits (10 eyes). For ex vivo studies, animals from each group were decapitated individually after intraperitoneal injection of 800 mg/kg sodium pentobarbital.

Corneas were carefully excised with the limbus preserved from enucleated rabbit eyes within 3–4 min of sacrifice. These specimens were mounted in silicone-diffusion chambers, with the epithelial surface oriented toward the donor compartment. Both the donor and receiver chambers were filled with 1 ml of phosphate-buffered saline (PBS, pH 7.4). Iontophoresis was administered using a constant current source with silver-silver chloride (Ag–AgCl) electrodes. The anode was positioned on the central corneal epithelium, and the cathode was placed in the receiver chamber. For clinical applications, current was applied at intensities of 0.5, 1, 2, 3, 4, 5, 6, 7, and for destructive non-clinical as a damage model 500 mA was applied. These currents are corresponding to current densities of 0.442, 0.884, 1.768, 2.653, 3.537, 4.421, 5.305, 6.190, and 442.1 mA/cm^2^, respectively, for duration of 10 s. The donor solution was supplemented with albumin at a concentration of 1 mg/ml. All experimental procedures were performed using independent samples (*n* = 10/group).

To ensure reproducibility of permeation conditions, corneas were mounted immediately after excision with the limbus preserved to maintain mechanical stability and minimize tissue leakage. The receiver chamber contained PBS maintained at a constant volume of 1 ml, and the donor chamber contained albumin solution (1 mg/ml) to maintain a large concentration gradient across the cornea. Given the short iontophoresis exposure time (10 s), the fraction of transported albumin remained small relative to the donor concentration, supporting sink-like conditions in the receiver compartment. Throughout experiments, the diffusion chamber was visually inspected for leakage or air bubble formation. All corneas were handled under consistent hydration conditions to minimize variability in permeability due to tissue dehydration.

### Determination of albumin concentration by fluorescence spectroscopy

Following the defined current application period of 10 s, receiver samples were collected for analysis. Fluorescence intensity was quantified using a spectrofluorometer (FS5, Edinburgh Instruments Ltd, Livingston, UK) equipped with a Xenon lamp. Measurements were performed in emission scan mode. Samples were excited at a wavelength of 280 nm, and emission spectra were recorded from 290 nm to 450 nm. Data were acquired at 10 nm intervals with the excitation and emission slit widths both set to 10 nm. A Suprasil quartz fluorescence cuvette with a 10 mm path length was used for all measurements. A sample volume of 700 µl of receiver fluid was used for each measurement, and albumin concentration was inferred from intrinsic tryptophan fluorescence.

To ensure quantitative reliability of intrinsic fluorescence measurements, albumin calibration standards were prepared in phosphate-buffered saline (PBS, pH 7.4) across a concentration range covering the expected permeation levels. A calibration curve was generated by plotting fluorescence intensity at the emission maximum (345 nm) against known albumin concentrations, and linear regression analysis -not constrained to pass through zero- was used to confirm linearity within the measurement range. All fluorescence measurements were performed under identical instrumental settings (excitation 280 nm, emission scan 290–450 nm, constant slit widths, identical cuvette path length) to minimize systematic variability. Each sample was measured in triplicate to confirm repeatability, and the mean intensity value was used for concentration estimation.

### Whole cornea Fourier-transform infrared spectroscopy

Corneas were initially stored at − 80 °C for 24 h, followed by freeze-drying under reduced pressure for an additional 24 h to remove residual free water from the tissue. The dried corneas were then mounted on a custom fixture featuring a 5-mm aperture, adapted from a manufacturer-supplied kit for KBr disks, to ensure uniform tissue spreading. Fourier-transform infrared (FTIR) spectroscopy was performed using a Nicolet iS5 spectrometer (Thermo Fisher Scientific Inc., USA) operating at room temperature. Spectra were acquired in absorbance mode with the infrared beam directed through the endothelial surface. The instrument was configured for a spectral resolution of 2 cm^−1^, and 250 scans were co-added for each measurement to optimize the signal-to-noise ratio. During data acquisition, the sample chamber was continuously purged with nitrogen gas to minimize atmospheric water vapor interference. Post-processing of spectral data was conducted as follows: a baseline correction was first applied using the instrument’s native software. Subsequently, spectra were smoothed using a 9-point Savitzky-Golay function. For each experimental group, individual spectra (*n* = 10 corneas) were averaged using OriginPro 2015 (OriginLab Corporation, USA) to generate a representative mean group spectrum for subsequent analysis. The averaged spectra were then subjected to second-derivative transformation using the instrumental software, with the resulting negative peaks displayed in the corresponding figures.

### Statistical analyses

All experimental data are presented as the mean ± standard deviation (SD) of independent corneal samples (*n* = 10). Data were compared across current groups using one-way analysis of variance (ANOVA) followed by Post-hoc analysis using Tukey’s honestly significant difference when analyzing the albumin concentration. A *p*-value < 0.05 was considered statistically significant. All analyses were performed using Origin Pro software 2015 (Origin Lab Corporation, Northampton, MA, USA).

## Results

### Corneal-simulated temperature distribution

Previous studies have established that tissue temperatures around 41 °C mark the onset of cellular stress and adaptive mechanisms (the “Caution” threshold), whereas temperatures exceeding 43 °C trigger irreversible thermal injury, defining the “Damage” threshold across ocular and other soft tissues^21^.

**Fig. 1:**
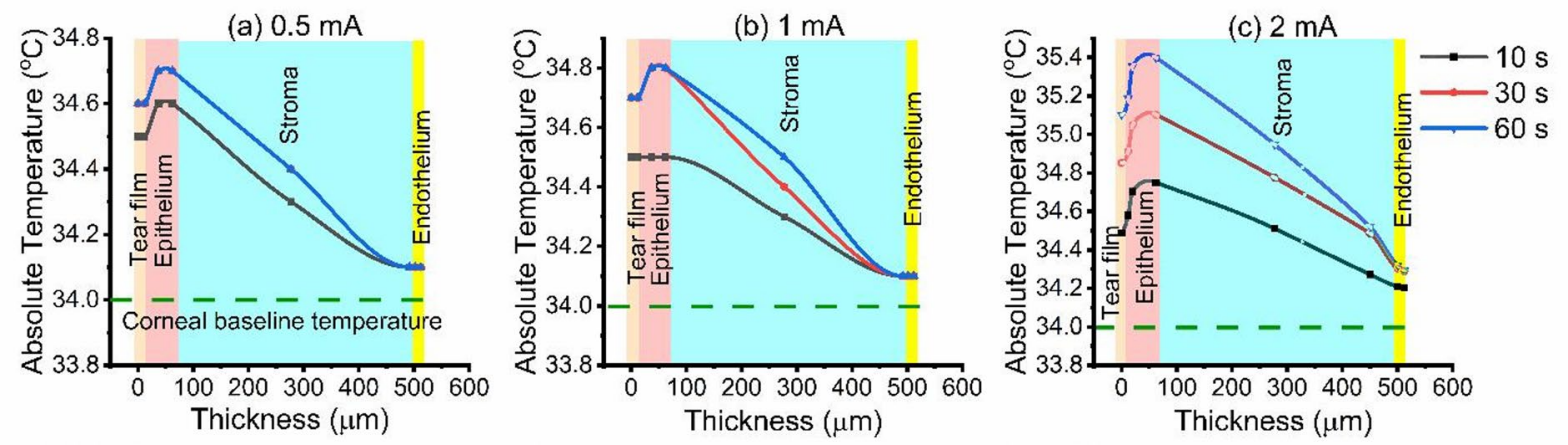
Temperature distribution through corneal layers during (**a**) 0.5 mA, (**b**) 1 mA, and (**c**) 2 mA iontophoresis at 10 s (black), 30 s (red), and 60 s (blue) exposure times.

**Fig. 2:**
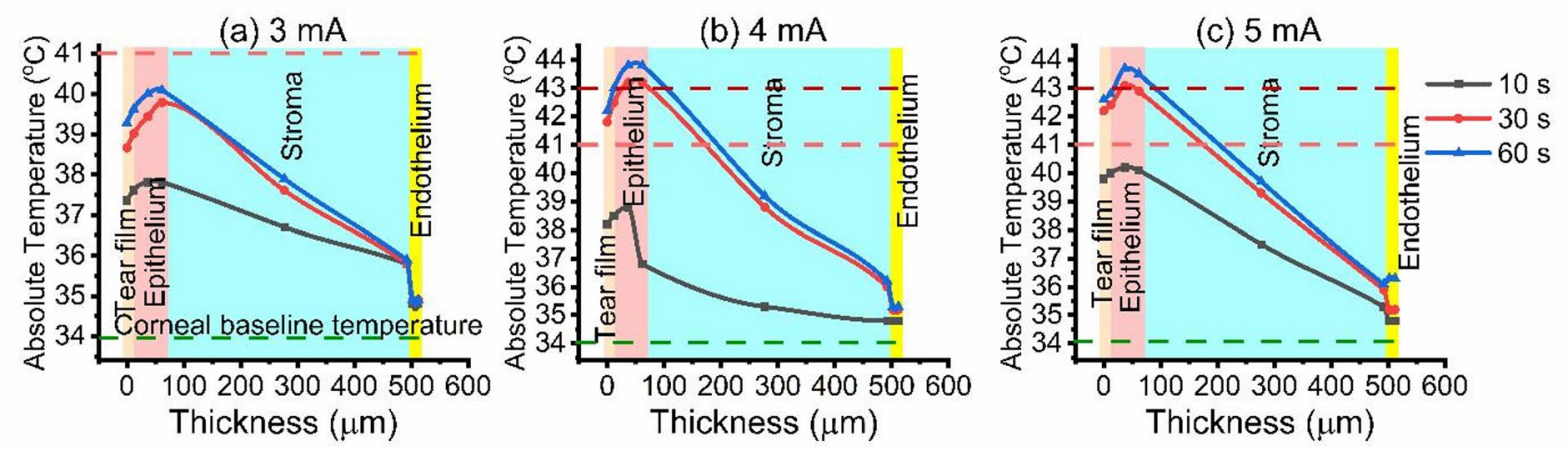
Temperature distribution through corneal layers during (**a**) 3 mA, (**b**) 4 mA, and (**c**) 5 mA iontophoresis at 10 s (black), 30 s (red), and 60 s (blue) exposure times.

Temperature elevation varied with current and duration, governed by Joule heating and corneal thermal diffusivity. In healthy conditions the temperature of the central cornea is lower than core body temperature, with reported mean values ranging between 32 and 36 °C^22,23^. Following 10 s of current exposure, distinct surface temperature response profiles were identified, corresponding to the applied current magnitude. At low currents (0.5–2 mA), corneal surface temperatures remained within the corneal baseline range (32–36 °C), indicating thermoneutral conditions (Fig. 1a–c). Increasing the current to 3 mA elevated the surface temperature to 37.3–37.6 °C (Fig. 2a).

**Fig. 3:**
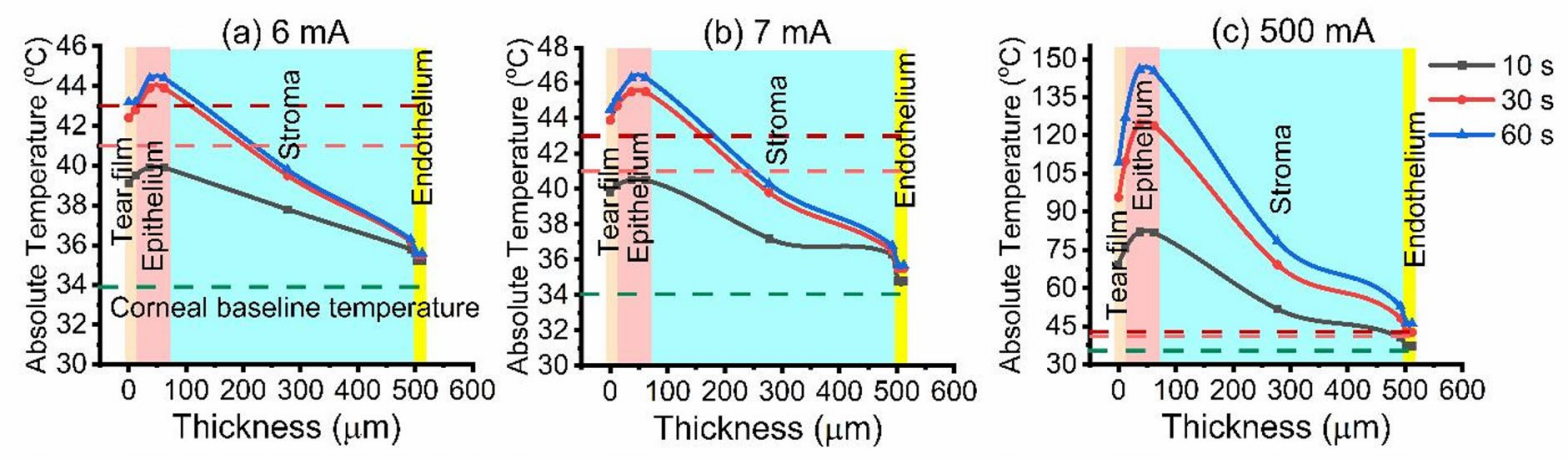
Temperature distribution through corneal layers during (**a**) 6 mA, (**b**) 7 mA, and (**c**) 500 mA iontophoresis at 10 s (black), 30 s (red), and 60 s (blue) exposure times.

Further increases to 4–7 mA induced more pronounced hyperthermia, with surface temperatures ranging from 38.3 to 40.5 °C as illustrated in Figs. 2 and 3. At the extreme stimulation of 500 mA, the model predicted a sharp rise in surface temperature to 82.1 °C, indicative of severe hyperthermia (Fig. 3c).

Increasing the iontophoretic time to 30–60 s, mild heating was observed at low intensities (0.5–2 mA), with surface temperatures of 34.1–35.4 °C. The tear film and epithelium exhibited the most rapid thermal response due to their high ionic conductivity and direct exposure to current, while the stroma, characterized by high water content and low thermal conductivity, showed a gradual depth-dependent thermal gradient. At these levels, the posterior stroma, and endothelium exhibited a non-damaging temperature increase of 0.1–0.2 °C, indicating safe energy transmission through the corneal layers. When the current was further increased to 3–7 mA, surface temperatures exceed the corneal temperature baseline, reaching 39.8–46.3 °C, and the anterior stroma entered the caution zone. Meanwhile, the posterior stroma and endothelium, although cooler, reached approximately 34.9–35.5 °C.

Under extreme iontophoretic stimulation (500 mA), simulated profiles predicted catastrophic heating, with the tear film exceeding 100 °C and epithelium reaching 146 °C after 60 s, conditions compatible with instant coagulative necrosis and tissue dehydration^24^. This prolonged exposure elevated the posterior stromal and endothelium temperatures to 48.2, and 42.7 °C respectively, indicating thermal stress.

**Fig. 4:**
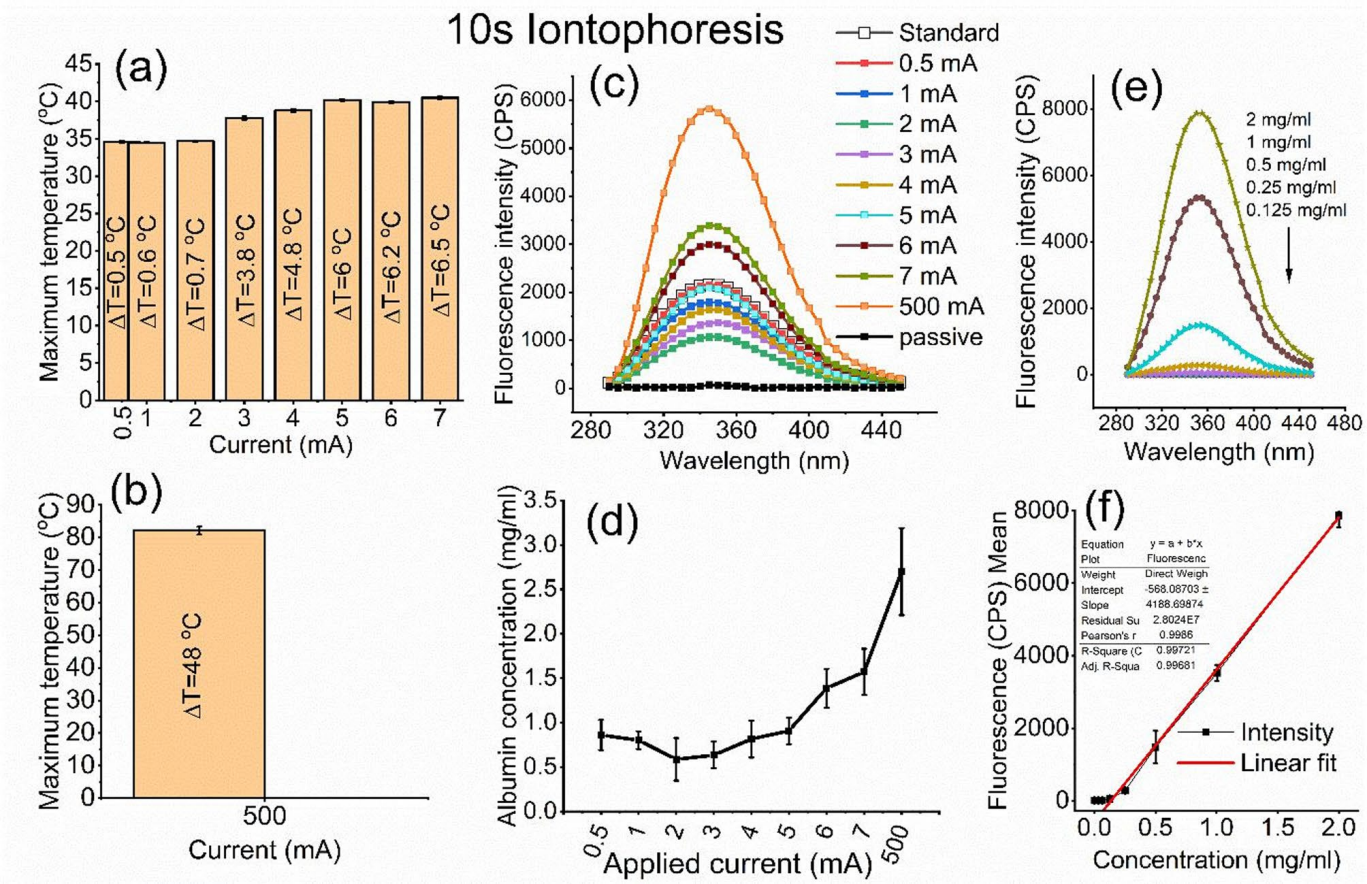
Representative histograms of maximum temperature at corneal surface (**a**, **b**) related to 0.5–500 mA applied currents, fluorescence spectra of albumin after transcorneal permeation at different current intensities (**c**), and the detected albumin concentration in the receiver chamber (**d**). The spectra of serially diluted albumin solution (**e**), and the corresponding standard curve (**f**) are also displayed.

As illustrated in Fig. 4a, the predicted maximum temperature and the temperature increase (DT) exhibits two distinct patterns; one remaining below the surface corneal threshold range at 0.5–2 mA, while the second pattern exceeds this threshold range at currents ≥ 3–7 mA. In contrast, a catastrophic increase in temperature is predicted at an applied current of 500 mA as shown in Fig. 4b.

### Albumin transcorneal transport

The efficiency of transcorneal iontophoresis in facilitating albumin delivery was quantitatively evaluated by measuring the fluorescence intensity of tryptophan moiety within the transported albumin across a wide range of applied electrical currents. Figure 4c presents the fluorescence emission spectra obtained following a 10-second iontophoretic application. Visually, the 0.5 mA spectrum resembled the standard albumin solution. A marked decrease in fluorescence intensity was observed as the current increased from 1 to 2 mA, followed by a progressive enhancement with further current increments from 3 to 500 mA. Notably, iontophoresis at 500 mA yielded a fluorescence intensity that surpassed all other conditions by several orders of magnitude. The passive transcorneal permeation of albumin was also included in the figure, and revealed a weak and broad intensity at 345 nm.

The albumin concentration presented in Fig. 4d illustrates the relationship between the applied iontophoretic current and the resulting albumin concentration in the receiver chamber following transcorneal transport. Albumin concentrations were quantified fluorometrically and normalized relative to a 1 mg/ml standard solution. The statistical analysis using ANOVA test with equal sample size across groups (*n* = 10) revealed a highly significant difference among the different current levels (*p* < 0.0001), indicating that the response was strongly dependent on the magnitude of the applied current. Post-hoc analysis using Tukey’s honestly significant difference (HSD) test revealed no statistically significant differences among applied currents ranging from 0.5 to 5 mA. In contrast, application of higher currents resulted in a marked and statistically significant increase in albumin concentration. Currents of 6 and 7 mA produced significantly higher concentrations compared with all lower current groups (≤ 5 mA; *p* < 0.01), while no significant difference was observed between the 6 and 7 mA groups themselves (1.7 mg/ml). The highest applied current (500 mA) exhibited the greatest concentration (2.7 mg/ml) and was significantly different from all other current levels (*p* < 0.001). Because intrinsic fluorescence is sensitive to the presence of other aromatic amino acid-containing proteins, interpretation of fluorescence measurements was considered most reliable under clinically relevant current ranges (≤ 7 mA), where structural tissue integrity was largely preserved. For the extreme non-clinical damage model (500 mA), fluorescence signals were interpreted cautiously, as barrier breakdown may release endogenous corneal proteins that can contribute to the measured emission signal.

Moreover, the emission spectra of the serially diluted albumin solution (Fig. 4e) and the corresponding calibration curve (Fig. 4f) demonstrated a linear detection range of 0.125–2 mg/ml.

### Vibrational characteristics of corneas

**Fig. 5:**
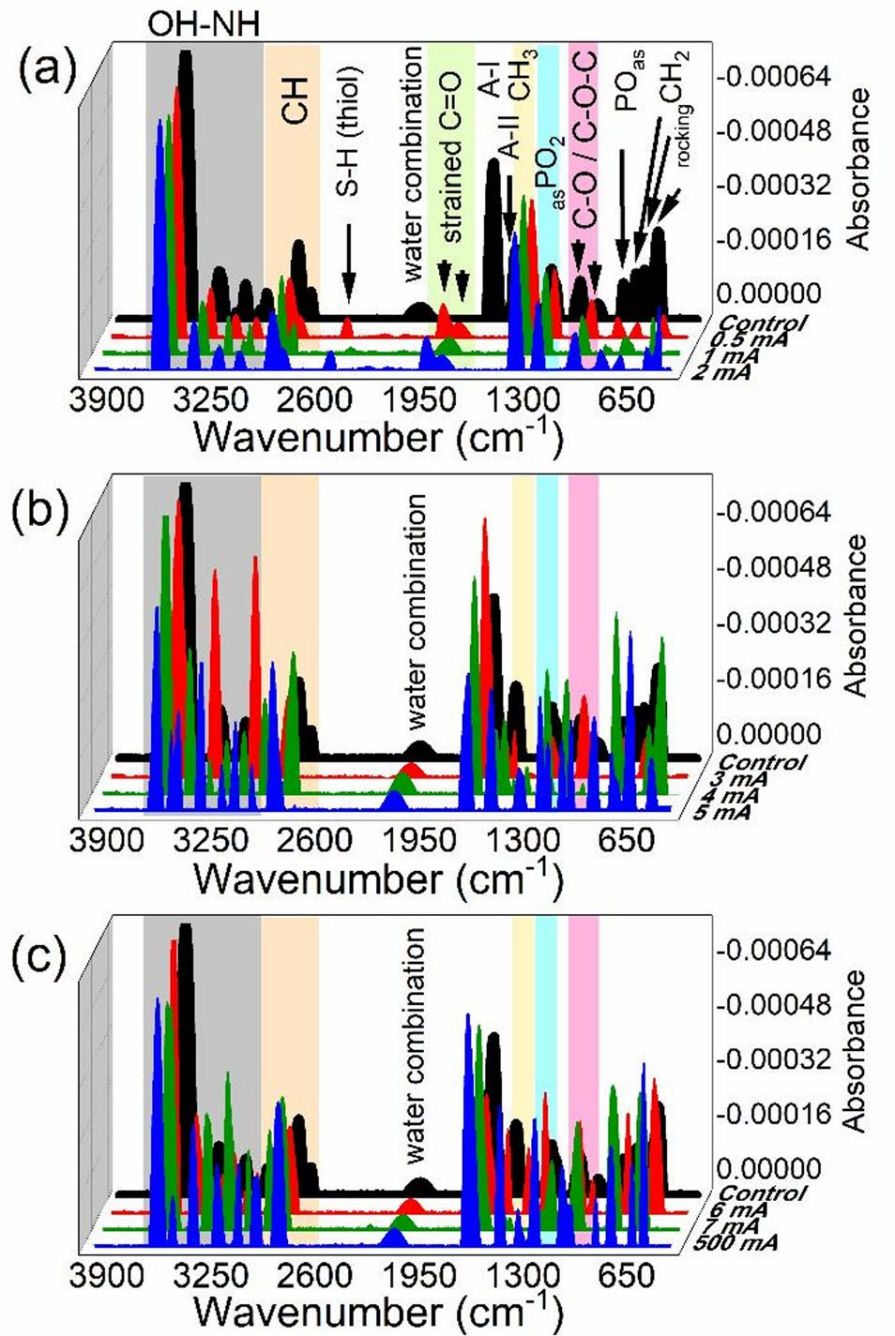
Collective FTIR second derivative spectra of whole corneas after exposure to different current intensities.

In the FTIR spectra (Fig. 5a–c), the band near 2129 cm^−1^ belongs to water molecules specifically a combination of the H–O–H bending (1640 cm^−1^) and O–H stretching (3400 cm^−1^) vibrations. Mathematically, this corresponds to: ${v}_{comb}={v}_{bend}+{v}_{stretch}$​. Even after lyophilization, bound water remains associated with biomolecules such as collagen (the dominant corneal protein), glycosaminoglycans in the stromal extracellular matrix, and hydrogen-bonded networks around amide, hydroxyl, and carboxyl groups. The persistence of the 2129 cm^−1^ band in freeze-dried cornea therefore indicates the presence of non-freezable, tightly bound water and structurally integrated within the macromolecular matrix. At low-moderate iontophoretic currents (0.5–2 mA) the 2129 cm^−1^ water combination band was detected at very-weak intensities. At higher currents (≥ 3 mA), stronger electrical effects enhance this water band.

FTIR spectroscopy revealed current-dependent spectral variations in the carbonyl and amide regions, which are commonly associated with protein conformational environment and hydration-related interactions. The control cornea exhibited a dominant amide I band centered near 1666 cm^−1^, which is consistent with stromal collagen and other structural proteins. Following iontophoresis, variations in peak positions and intensities were observed across groups, suggesting that electric field exposure and Joule heating may alter hydrogen bonding interactions, local hydration shells, and the vibrational coupling of peptide carbonyl groups. Although amide I band shifts are frequently used as indicators of protein secondary structural tendencies, FTIR alone cannot definitively resolve exact structural transitions without complementary methods such as circular dichroism or Raman spectroscopy. Therefore, the observed spectral shifts in the present study are interpreted as evidence of current-dependent protein conformational perturbation rather than direct proof of specific structural rearrangements. These interpretations are consistent with established FTIR protein structural analysis limitations^25,26^.

In contrast, corneas exposed to low-intensity iontophoretic currents (0.5–2 mA) displayed two additional absorption bands at 1929 cm^−1^ and 1821 cm^−1^. At higher iontophoretic current intensities (3–500 mA), the FTIR spectra showed a marked disappearance of the high-frequency carbonyl bands previously observed at 1929 cm^−1^ and 1821 cm^−1^, which were attributed to strained dicarbonyl structures formed at low current levels. Instead, the spectra were dominated by frequency changes within the amide I region (1600–1700 cm^−1^). At 3–5 mA, the predominant amide I component was detected at 1666–1682 cm^−1^, corresponding to β-turn conformations. Further increases to 6–7 mA produced a distinct shift to 1659 cm^−1^, characteristic of α-helical conformations. At the highest current intensity (500 mA), the detected amide I band at 1674 cm^−1^ is dominated by β-turn structures.

In IR spectroscopy, carbonyl vibrations in the 1800 cm^−1^ region can sometimes be associated with anhydride-like or strained carbonyl environments; however, the complexity of biological tissue composition and overlapping vibrational contributions limits definitive molecular assignment. Therefore, while these peaks may reflect current-induced modifications in carbonyl-containing biomolecules or localized chemical microenvironments, they should be interpreted cautiously. Similar spectral regions have been discussed in polymeric and anhydride formation studies, but further validation would be required to confirm the exact biochemical origin in corneal tissue^27,28^.

In the control corneal tissue, the FTIR spectrum exhibited amide II absorption bands centered at 1527 cm^−1^, consistent with typical protein backbone conformation. Upon iontophoresis at 3 mA, this band upshifted to 1535 cm^−1^. At 4 mA, a further upward shift was observed, with amide II at 1551 cm^−1^. Moreover, when the current was increased to 5–6 mA, amide II frequency remained unchanged. At 7 mA, the band was decreased in frequency; 1512 cm^−1^. Finally, under 500 mA, the amide II band was undetected. Table 3 (supplementary material) summarizes these frequency variations and their significance as well.

Following iontophoretic treatment at low current intensities (0.5–2 mA), the FTIR spectra of corneal tissue also revealed the emergence of a weak but distinct absorption band within the 2450–2530 cm^−1^ region, corresponding to the S-H stretching vibration of thiol groups. This band was absent in the control spectra as well as at higher currents (3–500 mA).

The FTIR-second derivative of the OH stretching region revealed distinct alterations in the integrated area percentages of the hydroxyl vibrational modes: stretching (_str_OH), asymmetric (_asy_OH), and symmetric (_sy_OH) as a function of iontophoretic current intensity as illustrated in Fig. 5a–c and extended Fig. 6a–d. These integrated areas represent the relative population of each vibrational component contributing to the total OH absorbance.

Under control conditions, the total integrated OH area was 38.1 ± 3.3%, dominated by the _str_OH contribution (31.1 ± 0.9) with smaller _asy_OH (4.7 ± 0.7%) and _sy_OH (2.2 ± 0.5%) fractions, indicating a predominance of tightly bound structural water. At low current intensities (0.5–2 mA), a common non-significant increase was observed that included the total OH area 42.4 ± 3.9% (0.5 mA), 41.8 ± 3.7% (1 mA), and 40.8 ± 4.2% (2 mA). This non-significant increase was extended to include _asy_OH (from 4.9 ± 0.7 to 5.9 ± 0.9%), _sy_OH (from 2.3 ± 0.1 to 2.4 ± 0.2%), and _str_OH (from 33.5 ± 0.19 to 34.9 ± 3.1%), reflecting non-significant molecular heterogeneity. At current levels of 3–500 mA, the total OH area markedly declined corresponding to a reduction in _str_OH and fluctuations of _asy_OH and _sy_OH components. Table 4 (supplementary material) summarizes the integrated area percentages of different OH vibrational modes.

**Fig. 6:**
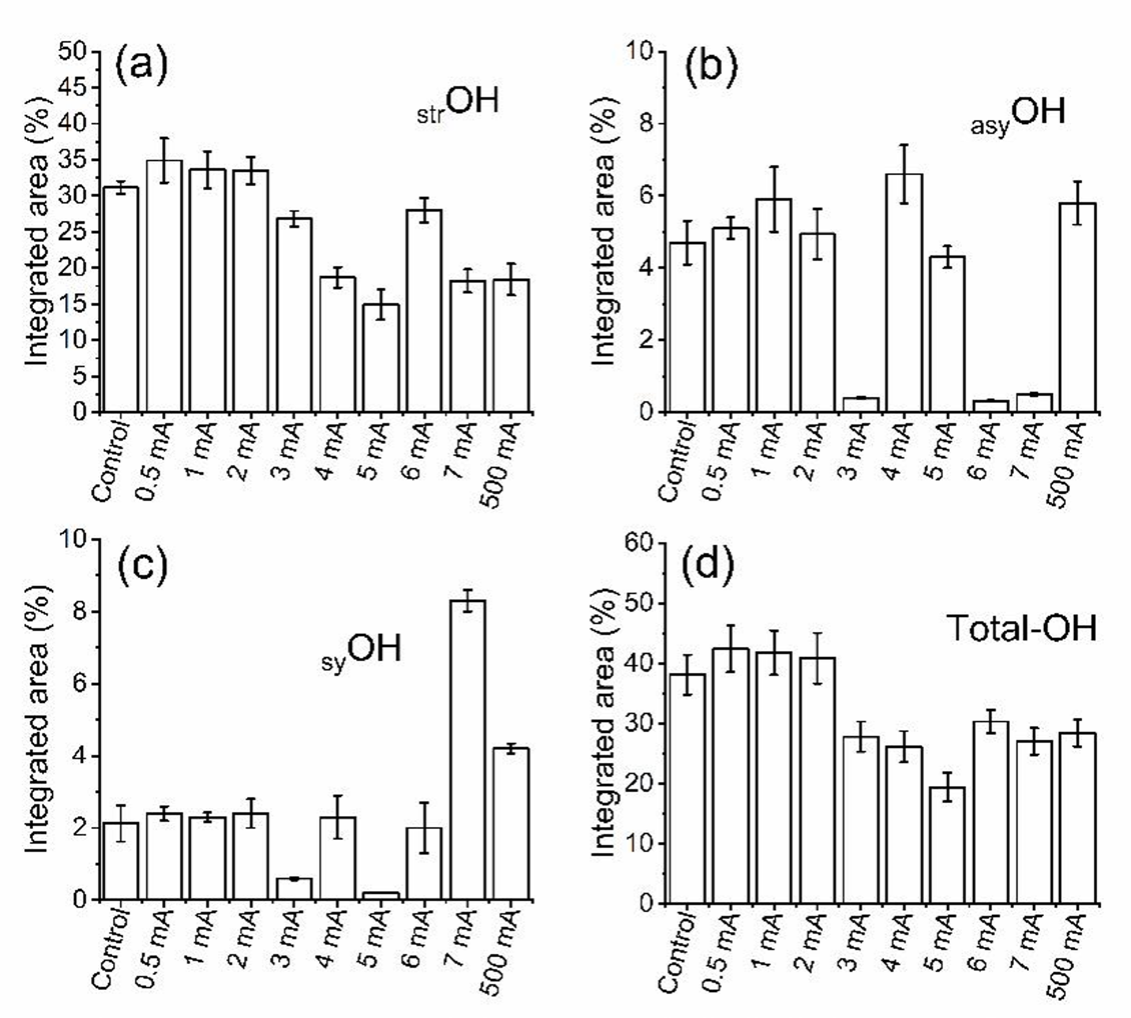
Integrated area percentage of (**a**) stretching OH, (**b**) asymmetric OH, (**c**) symmetric OH, and (**d**) total OH area after iontophoresis with different current intensities.

**Fig. 7:**
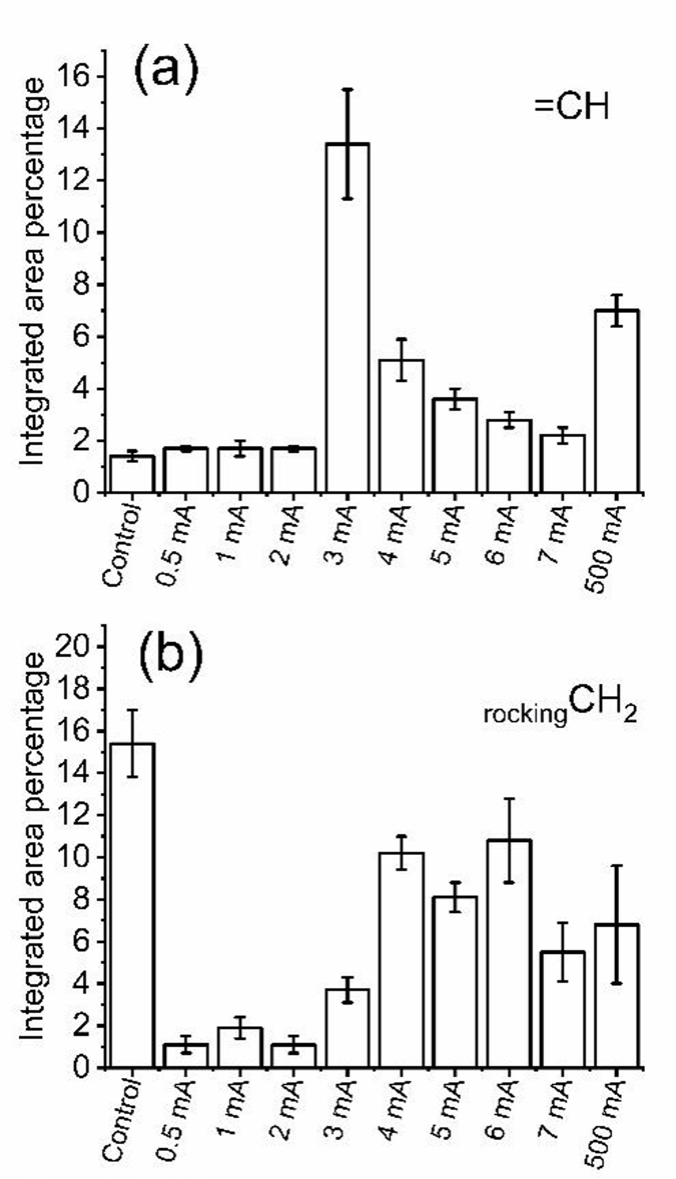
Integrated area percentage of (**a**) unsaturated CH (=CH), and (**b**) the rocking CH_2_ modes of vibrations.

As shown in Fig. 7a, the integrated area percentage of the unsaturated CH (=CH) band remained comparable to the control following exposure to 0.5–2 mA, but exhibited a significant increase at currents ≥ 3 mA. In contrast, Fig. 7b, illustrating the rocking CH_2_ vibrational mode, demonstrates a consistent decrease across all applied currents, with the most pronounced reduction observed within the 0.5–2 mA range.

## Discussion

Transcorneal iontophoresis utilizes electric current to facilitate drug delivery; however, the accompanying Joule heating poses a considerable risk to tissue integrity. Thus the thermal thresholds “Caution” and “Damage” are delineate the transition from reversible metabolic stress (41 °C) to rapid, irreversible coagulative necrosis (≥ 43 °C) as protein denaturation and cell death accelerate exponentially with temperature^29^. In the present context, experimental findings in ocular tissues corroborate these limits, where porcine corneas exposed to ≤ 42 °C for 12 h retained endothelial integrity, whereas temperatures exceeding 42 °C induced measurable cell loss^30^. Likewise, retinal studies by Brinkmann et al. demonstrated minimal injury below 40 °C, increasing sharply beyond 43 °C^31^.

Clinically, these principles form the basis of thermal-based ophthalmic interventions. For example, laser thermokeratoplasty achieves controlled stromal collagen shrinkage at temperatures of approximately 60–65 °C. In contrast, non-ablative modalities such as ocular iontophoresis require strict regulation of corneal temperature, which must be maintained below 43 °C and preferably under 41 °C to preserve cellular integrity and ensure tissue viability^32^. Therefore, it is important to distinguish the ocular safe temperature margin (32–36 °C) from other tissue physiologically safe margin (41–43 °C). Accordingly, the present results indicate that currents ≤ 2 mA satisfying both thermal safety margins, while iontophoresis with 3 mA satisfying only the physiological safety margin. In the same context, increasing the current to 4–6 mA raise the induced thermal burden to the caution margin after 10 s exposure. Further current exposure at 30–60 s drive the thermal burden to the damage zone with surface temperature exceeded 80 °C at 500 mA, confirming a complete departure from the therapeutic safety window.

Note that, the computational thermal model applied in this work is based on the Pennes bioheat equation, which has been widely used for predicting heat transport in biological tissues, including ocular structures. The model incorporates tissue thermal properties and Joule heating under applied current, enabling estimation of current-dependent corneal temperature elevation. Nevertheless, direct experimental temperature measurements (e.g., infrared thermography or micro-thermocouple recordings) were not performed in the present ex vivo setup. Therefore, while the model provides mechanistically reasonable estimates of thermal behavior and safety thresholds, experimental validation represents an important future step to strengthen quantitative correlation between predicted heating and biological outcomes.

### Ocular and physiologically tolerable range (0.5–3 mA)

At 0.5–2 mA, rapid heating of the tear film and epithelium, followed by slower stromal propagation, reflects the cornea’s anisotropic thermal conductivity and layered hydration profile. The anterior regions, rich in water and metabolic activity, efficiently absorbed and dissipated heat, while posterior layers exhibited the minimum temperature rise consistent with conductive diffusion. Therefore, iontophoresis at this current range maintaining corneal viability and bioheat stability suitable for therapeutic applications^33^. Although thermal burden due to application of 3 mA fails within the physiological-safety limit, comparable findings in ocular hyperthermia studies confirm that temperatures between 38 and 40 °C enhance cellular activity, permeability, and drug transport without compromising corneal integrity^31^ by modulating metabolic and enzymatic activity via Q_10_-dependent acceleration^34^ and transient activation of thermosensitive TRP channels (TRPV1, TRPV4)^35^.

### Cautious, damage and ablative thermal kinetics (4–500 mA)

Currents of 4–7 mA produced physiological temperatures at the surface surpassing the damage threshold for coagulative necrosis and protein aggregation^36^. Such conditions are associated with epithelial erosion, stromal collagen degradation, and nociceptive activation through TRPV1 channels^35^ compromising barrier and optical properties, although posterior stroma and endothelium regions remained within the ocular safety limit. Destructive thermal kinetics with surface temperatures of 125–146 °C were achieved at 500 mA causing instantaneous protein coagulation and collagen collapse due to irreversible thermal denaturation^37,38^. These extreme responses indicate complete loss of corneal function and transparency. Moreover, the extreme Joule heating at 500 mA can lead to vaporization and structural ablation of the tear film and epithelium^39,40^. Although stromal temperatures declined exponentially with depth due to high water content and thermal diffusivity^41^, the anterior stroma still exceeded 41 °C, suggesting risk of transient stress responses such as heat-shock protein induction^37,38^. The corneal endothelium remained below the critical 43 °C threshold but approached sublethal stress levels (42.7 °C) posing potential risk to pump function and hydration balance^35,36^.

### Transcorneal permeation of albumin

The findings from this study indicate that iontophoresis for 10 s enhances albumin transport relative to the passive diffusion, and albumin transport due to currents of 0.5 mA to 5 mA is a hallmark of iontophoretic delivery. In the same context, currents of 6–7 mA resulted in accumulation of albumin. This phenomenon is primarily governed by electrorepulsion (electromigration) for charged molecules and electroosmosis flow induced by the current. The cornea is negatively charged at physiological pH; therefore, the application of an anodal current generates an electroosmotic flow from the anode to the cathode, which can carry neutral and positively charged species across the tissue^6^. While albumin is negatively charged at neutral pH (its isoelectric point is 4.7), its transport due to the applied currents is dominated by electroosmotic flow, which can overcome its electrophoretic mobility, and by possible current-induced alterations to the corneal tissue integrity. The low-ocular safe currents (0.5–2 mA) were associated with dependent-decrease in the observed albumin concentration, where the induced thermal burden (ΔT = 0.5–0.7 °C) remaining within the normal corneal temperature range (32–36 °C) which can be attributed to dominance of electroosmotic counterflow and limited electromigration for large macromolecules, which can hinder effective advancement of charged proteins across the corneal barrier. In biological membranes and tissues, electroosmotic flow produces bulk solvent motion in the direction of counterion flow that can either assist or impede protein transport depending on tissue charge density and permeant characteristics, particularly for macromolecules with low charge density such as albumin^42^. In such case, corneal barrier integrity is largely preserved, and the thermal energy is insufficient to significantly increase paracellular permeability or disrupt albumin-tissue interactions^14^. As the applied current increased to 3–5 mA, where thermal burden within the physiologically-safe limit and ΔT = 3.8–6 °C, albumin concentration was proportionally increased as the current increased due to enhanced electrical field strength and mild Joule heating, thereby facilitating macromolecular transport. At higher currents (6–7 mA), although the induced thermal burden fall within the physiologically tolerable limit, the continued increase in transcorneal albumin transport -i.e. the accumulation of albumin- likely reflects synergistic effects of elevated electric field, and relaxation of binding interactions within the extracellular matrix that collectively reducing steric and electrostatic barriers to protein movement^43^.

The dramatic increase in albumin concentration observed at 500 mA suggests a breakdown of the primary active transport mechanisms. At this supra-physiological current, the integrity of the corneal epithelial barrier is compromised due to extensive increase in ΔT (48 °C). This is consistent with studies showing that iontophoresis can disrupt tight junctions and alter tissue structure^44^. The concentration detected at 500 mA, being significantly higher than equivalent standard solution, implies not only loss of the barrier function, but also would be associated with significant tissue damage that led to the release of intracellular proteins.

It should be noted that the intrinsic fluorescence emission of albumin, with a maximum intensity (λ_max_) observed at 345 nm (Fig. 4c), demonstrated minimal spectral perturbation following 10 s of transcorneal iontophoresis across all tested current intensities. This spectral stability indicates the preservation of albumin’s native structure throughout iontophoretic procedure. The emission signal, originating primarily from tryptophan residues, is a sensitive probe of protein conformation; its characteristic λ_max_ near 345–350 nm is diagnostically responsive to changes in local polarity and packing^45^.

### Evaluation of corneal structure

FTIR spectroscopy provided detailed molecular insight into the corneal structural responses under different iontophoretic current intensities. Characteristic vibrational bands corresponding to water, amide, and thiol groups were evaluated to assess hydration dynamics, protein secondary structure stability, and redox-related changes, all of which reflect the molecular integrity of the corneal matrix. It should also be noted that corneas were freeze-dried prior to FTIR acquisition to reduce spectral interference from bulk water and to improve reproducibility of absorbance features. While lyophilization is commonly applied in IR spectroscopy of biological tissues, dehydration can influence hydrogen bonding networks and may shift the positions of amide and hydroxyl-related bands. Therefore, FTIR results primarily reflect relative differences between treatment groups under identical sample preparation conditions, rather than absolute hydration levels in native tissue. This limitation is consistent with established FTIR protein spectroscopy considerations^25,26^.

The water combination band at 2129 cm^−1^, is highly sensitive to hydrogen-bond strength and molecular packing. This bound water plays a critical role in maintaining the collagen triple-helix conformation, interfibrillar spacing and transparency, and the hydration shells surrounding proteoglycans that maintain tissue transparency and mechanical resilience. In addition, its intensity and frequency are sensitive to changes in the rigidity and connectivity of hydrogen bonds in the aqueous environment and to structural perturbations^46,47^.

Bulk water is characterized by a dynamic and disordered structure, facilitates molecular movement. It also introduces dynamic flexibility, facilitating conformational changes and molecular mobility while supports the dynamic rearrangements needed during folding intermediates or allows substrates to access and exit enzyme active sites. Moreover, it provides the necessary fluidity and nanosecond-scale flexibility for processes like vesicle formation and membrane fusion^48,49^. The contradictory findings regarding water band indicates that water molecules undergo redistribution near collagen binding sites, and the temperature simulation results given in Fig. 4a,b indicates that this redistribution is sensitive to ΔT. At low applied currents (0.5–2 mA), the reduced band intensity can be interpreted as a field-induced ordering/confinement of bulk water into more structured hydration states thereby diminishing the spectroscopic signature of loosely hydrogen-bonded water^50,51^. As the current increases (3–7 mA), Joule heating weakens hydrogen bonds, promoting a partial conversion of structured hydration water to less constrained, more bulk-like water, which increases the combination band intensity. With extreme current (500 mA), catastrophic thermal burden (Δt = 48 °C) further liberate water molecules from ordered networks into disordered, bulk-like states, also yielding enhanced band intensity. Such behavior is consistent with the temperature dependence of water hydrogen-bond connectivity, where increased thermal energy weakens the network, shifting spectral features and increasing the infrared absorption of combination bands as hydrogen bonds break and reform dynamically^47^.

The emergence of distinct high-frequency carbonyl absorptions at 1929 cm^−1^ and 1821 cm^−1^ in corneal tissue subjected to low-current iontophoresis (0.5–2 mA) represents a noteworthy spectral deviation from the conventional amide I region (1600–1700 cm^−1^) typically associated with unmodified peptide C=O stretching vibrations. The occurrence of carbonyl bands at such elevated wavenumbers indicates the formation of strained, non-peptidic carbonyl functionalities, most plausibly cyclic anhydrides, rather than conventional amide structures. Infrared spectroscopic analyses have established that acid anhydrides exhibit two characteristic carbonyl stretching modes: for acyclic anhydrides, the asymmetric and symmetric C=O stretching vibrations appear near 1819–1820 cm^−1^ and 1750 cm^−1^, respectively^27,28^. Acyclic anhydrides play an important role in maintaining corneal health and transparency through their influence on tissue hydration and structural organization. Corneal transparency is primarily governed by the highly ordered arrangement of collagen fibrils, a structure finely regulated by proteoglycans and stromal hydration levels. By modulating metabolic processes within corneal cells -particularly in the endothelium, which actively controls corneal hydration- acyclic anhydrides could contribute indirectly to the maintenance of optimal water balance essential for transparency^52,53^. In addition, acyclic anhydrides can influence the synthesis, cross-linking, and spatial organization of collagen fibrils, thereby supporting the biomechanical stability and optical clarity of the cornea^54^. This observation supports the hypothesis that mild electrochemical conditions during low-current iontophoresis promote localized intramolecular condensation or crosslinking reactions, yielding transient or stable strained carbonyl species within the corneal extracellular matrix.

To analyze protein secondary structure using FTIR spectroscopy, the amide I absorption component frequency correlates with distinct secondary structural motifs: β-turns are typically associated with bands above 1660 cm^−1^, α-helices with bands near 1650–1659 cm^−1^ and other motifs such as β-sheets and random coil at lower frequencies^55^. In the control cornea, the amide I maximum at 1666 cm^−1^ primarily reflects β-turn and loop structures that dominate the native protein structure. Upon iontophoretic at currents of 3–5 mA, electric field and thermal perturbation can transiently destabilize weaker hydrogen bonds without extensive backbone rearrangement, thereby maintaining β-turn dominance within the spectral range characteristic of this motif. As current increases further (6–7 mA), Joule heating and field effects preferentially disrupt turn-associated hydrogen bonds, promoting formation/stabilization of α-helical conformations reflected by a shift toward 1659 cm^−1^; similar helix enrichment under external perturbations has been observed in studies of protein response to thermal and electromagnetic stimuli^56–58^. At extreme current (500 mA), the reversion to β-turn may be indicative of irreversible damage, protein unfolding, and reorganization driven by overwhelming thermal stress on the corneal proteome, consistent with models of heat-induced conformational changes^59^.

The position and intensity of the amide II band during corneal iontophoresis reflect current-dependent modifications in N-H bending and C-N stretching vibrations, which are highly sensitive to hydrogen-bonding strength, peptide backbone orientation, and protein-water interactions rather than to secondary structure alone. Compared with the amide I band, the amide II band is intrinsically weaker and more susceptible to spectral overlap or suppression, rendering it particularly sensitive to conformational state and local environmental conditions^60–62^. In native corneal tissue, the amide II band detected at 1527 cm^−1^, and the preservation of comparable band characteristics at low applied currents (0.5–2 mA) indicates that mild electric fields do not substantially perturb peptide N-H interactions or proton exchange dynamics. At moderate currents (3–4 mA), the pronounced upshifts to 1535 and 1551 cm^−1^ can be attributed to reorientation of N-H hydrogen bonds, reduced hydrogen-bond donation to surrounding water molecules, and increased backbone rigidity induced by the combined effects of electric-field and localized Joule heating. Such blue shifts are well documented in FTIR studies of proteins undergoing partial dehydration or alterations in hydrogen-bond geometry^25,26,63^. The subsequent disappearance of the amide II band at 5–6 mA reflects strong band broadening and loss of vibrational coherence, arising from increased conformational heterogeneity, and disruption of peptide-water coupling^64^. At 7 mA, the reappearance of the band at a markedly lower frequency (1512 cm^−1^) indicates strengthening of N-H hydrogen bonding and increased backbone flexibility, consistent with partial unfolding, exposure of peptide groups to water, and enhanced proton mobility^26^. Moreover, the complete loss of the amide II band at extreme current (500 mA) where protein backbones lose ordered secondary structure entirely, and the characteristic vibrations of N–H bending and C–N stretching cannot be resolved, is indicative of irreversible protein denaturation, and backbone disorder due to thermal burden.

The transient appearance of a thiol (S–H) band in the 2450–2530 cm^−1^ region during corneal iontophoresis at low applied currents (0.5–2 mA), followed by its disappearance at higher currents (≥ 3 mA), reflects current-dependent redox, exposure, and environmental effects on cysteine residues. In native corneal tissue, thiol groups are largely buried within protein interiors or engaged in disulfide bonds (-S-S-) ^26,65,66^, and the S-H stretching vibration is intrinsically weak; consequently, it is often undetectable in control FTIR spectra due to low oscillator strength and overlap with background absorption. At low iontophoretic currents, mild electric-field polarization and subtle protein conformational relaxation may transiently expose reduced cysteine residues or promote partial reduction of disulfide bonds, allowing free –SH groups to become spectroscopically detectable within the characteristic 2450–2530 cm^−1^ range. This is consistent with reversible structural adaptation without significant thermal or oxidative stress^67^. The presence of these thiol signals indicates increased availability of reduced sulfur-containing species, including cysteine and glutathione, implying activation of thiol-dependent antioxidant pathways and redox-sensitive metabolic responses^68,69^.

As the applied current increases beyond 3 mA, the disappearance of the thiol band is attributed to oxidation of free thiols to disulfides, strong hydrogen bonding with surrounding water, or extensive conformational disorder, all of which suppress the S-H stretching vibration beyond detectability^70,71^. At extreme current (500 mA), irreversible protein denaturation and oxidative damage driven by increased ΔT further eliminate coherent thiol signatures by driving cysteine residues into non-native states, aggregated structures, or chemically modified forms.

The current-observed variations in the integrated areas of total OH, stretching OH, asymmetric OH, and symmetric OH bands during corneal iontophoresis reflect non-linear reorganization of the corneal hydrogen-bonded water network. At low applied currents (0.5–2 mA), the absence of significant changes in OH-related integrated areas relative to the control indicates preservation of physiological hydration and hydrogen-bonding architecture^10,72^. In this context, these electric fields are insufficient to perturb bulk water populations or disrupt protein-water interactions, and electroosmotic water movement is compensated by rapid redistribution within the tissue, resulting in spectrally invariant OH signatures. As the applied current increases to 3–7 mA, the observed reduction in total OH and stretching OH integrated areas can be attributed to increased ordering of water molecules into structured hydration shells, coupled with Joule heating that weakens intermolecular hydrogen bonds and alters vibrational coupling. At the extreme, non-clinical current of 500 mA, further reductions in total and stretching OH integrated areas are consistent with thermal stress and irreversible tissue disruption, including collapse of organized hydration layers, and partial sequestration of water into non-IR-active environments. Comparable reductions in OH absorbance have been reported in dehydrated or denatured tissues, where the breakdown of extended hydrogen-bond networks leads to collagen compaction and reduced corneal permeability^73–75^. Accordingly, the altered balance between asymmetric and symmetric OH bands reflect a breakdown of coherent hydrogen-bond networks and increased heterogeneity in water-protein interactions. These findings demonstrate that OH band behavior during iontophoresis is governed by current-dependent hydration restructuring and thermal effects.

The current-dependent changes noticed in the integrated area percentage of the unsaturated CH(=CH) band during corneal iontophoresis reflect alterations in lipid conformational order, and membrane packing. The stability of the =CH band following exposure to low applied currents (0.5–2 mA) indicates preservation of native lipid organization and membrane integrity, suggesting that these electric fields do not substantially perturb unsaturated acyl chain conformations or expose additional olefinic CH groups to the infrared probing volume. In contrast, the significant increase in the =CH band area observed at currents of 3–7 mA indicates an increased membrane fluidity, trans-gauche isomerization of unsaturated fatty acyl chains, and relaxation of lipid-protein interactions that enhance the vibrational freedom of olefinic groups. FTIR studies of biological membranes have shown that such increases in lipid disorder and phase fluidization are accompanied by enhanced intensities of unsaturated CH stretching modes, even in the absence of compositional changes^76–78^. At the extreme, non-clinical current of 500 mA, the further elevation of the =CH band area likely reflects severe membrane disruption and oxidative stress, including breakdown of tightly packed lipid domains, which increase the relative contribution of disordered and oxidized unsaturated lipid species to the FTIR spectrum^79^. These observations identify the unsaturated CH band as a sensitive spectroscopic indicator of current-induced membrane perturbation, distinguishing reversible lipid fluidization at moderate iontophoretic currents from irreversible membrane damage at extreme electrical loading.

Finally, the rocking vibrational mode of CH_2_ groups within the lipid chains of cell membranes is related to the packing and phase transitions of these lipids. This molecular motion is sensitive to the physical state of the cell membranes and thus plays a role in maintaining the structural integrity and function of the cornea’s cellular layers^80,81^. In native corneal tissue, the presence of this band reflects relatively ordered lipid domains within epithelial and stromal cell membranes. The consistent reduction in the intensity of the CH_2_ rocking vibrational mode relative to the control across all applied currents indicates alterations in lipid chain packing and lateral ordering. The pronounced decrease observed even at low applied currents (0.5–2 mA) suggests that the electric-field exposure is sufficient to affect lateral lipid packing, which induces subtle increases in lipid fluidity, and the reduction of coherent CH_2_ rocking vibrations. The comparable attenuation observed at currents of 3–7 mA implies that once a threshold level of lipid disorder is reached, further increases in current do not proportionally enhance lateral packing, consistent with a saturable disordering process. These findings suggesting that currents (0.5–7 mA) enhance albumin permeability by transiently modifying lipid organization without structural damage^82^. At the extreme non-clinical current of 500 mA, and due to thermal stress, the continued suppression of the CH_2_ rocking mode is attributed to membrane disorganization due to extensive trans-gauche isomerization, and phase mixing^83^.

The clinical translation of corneal iontophoresis for HMW therapeutics is promising but faces substantial regulatory, manufacturing, and practical challenges. As drug-device combination products, iontophoretic systems must satisfy dual regulatory requirements under agencies such as the U.S. Food and Drug Administration and the European Medicines Agency, necessitating rigorous demonstration of pharmacological safety, electrical performance, current-density reproducibility, and long-term ocular tolerability. For biologics, additional analytical validation is required to confirm that electrical exposure does not induce structural instability, aggregation, or altered immunogenicity.

From a manufacturing standpoint, ensuring batch-to-batch consistency of HMW therapeutics must be coupled with strict standardization of electrode materials, geometry, and tissue contact to achieve reproducible dosing and controlled thermal profiles. Economically, development costs exceed those of conventional topical formulations due to device engineering, quality assurance, and regulatory compliance. Clinically, successful adoption depends on practitioner training, patient tolerability, workflow integration, and robust randomized trials demonstrating safety and efficacy relative to established interventions. Addressing these multidimensional barriers is essential for the successful translation of corneal iontophoresis into routine ophthalmic practice.

## Conclusions

This integrated experimental and computational study demonstrates that transcorneal iontophoresis can enhance the delivery of HMW macromolecules such as albumin across excised rabbit corneas. Computational thermal modeling indicated that low iontophoretic currents (≤ 2 mA) maintain corneal surface temperature within the physiological ocular range (32–36 °C), supporting an ocular-safe operational window for macromolecular delivery. Moderate currents (3–7 mA) produced progressively higher thermal elevations, suggesting that exposure duration becomes critical in preventing supraphysiological heating and potential thermal stress. Fluorescence quantification confirmed enhanced albumin transport relative to passive diffusion, with significantly increased permeation at 6–7 mA. FTIR spectroscopy revealed current-dependent molecular alterations consistent with changes in hydration-related vibrational features, protein conformational environment, and lipid organization, indicating reversible adaptation at low-to-moderate currents and pronounced disruption under extreme exposure. The extreme non-clinical current (500 mA) produced catastrophic heating and barrier breakdown, confirming that excessive electrical loading leads to irreversible tissue damage. Collectively, these findings highlight that careful optimization of current intensity and application time is essential to maximize transcorneal delivery of biologics while preserving corneal integrity, supporting the translational potential of iontophoresis as a non-invasive ocular delivery platform.

### Limitations and future perspectives

Although this study provides integrated experimental and computational evidence supporting transcorneal iontophoresis as a promising platform for macromolecular delivery, several limitations should be acknowledged. First, thermal modeling was not directly validated by experimental temperature measurements during iontophoresis. Future studies incorporating real-time corneal thermography or embedded micro-thermocouple measurements would provide stronger validation of the predicted Joule heating profiles. Second, fluorescence-based albumin quantification relies on intrinsic aromatic emission and may be influenced by endogenous protein release in groups where severe tissue disruption occurs, particularly under extreme non-clinical current exposure. Future work may include complementary quantification methods such as Bradford assay, ELISA, or SDS-PAGE to confirm albumin specificity. Third, FTIR spectroscopy provides sensitive molecular-level information but does not uniquely resolve exact biochemical structures, and therefore interpretations regarding protein conformational changes and hydration network rearrangement should be considered indicative rather than definitive. Additional validation using Raman spectroscopy, circular dichroism, or histological assessment would strengthen structural conclusions. Finally, this work was performed under ex vivo conditions using rabbit corneas, and in vivo translation requires consideration of tear turnover, blinking, epithelial repair mechanisms, and ocular discomfort. Future investigations should explore clinically relevant biologic therapeutics to define safe and effective protocols suitable for translation into ophthalmic practice.

## Supplementary Information

Below is the link to the electronic supplementary material.

Supplementary Material 1

Supplementary Material 2

Supplementary Material 3

## Data Availability

The data are available within the article and Supplementary Material.
