# Supplementary material for: Ex vivo and computational investigation of corneal iontophoresis to enhance penetration of high-molecular-weight compounds: a study using albumin as a model molecule
Source: Sci Rep. 2026 Mar 31;16:10990. doi: 10.1038/s41598-026-43580-y (PMC13043707; doi:10.1038/s41598-026-43580-y)
Supplement: Supplementary file 2 — Supplementary Material 2 [file 41598_2026_43580_MOESM2_ESM.docx]

Table 3. FTIR band assignments and structural interpretation of corneal proteins after iontophoresis

| Current (mA) | Amide I (cm^-1^) | Amide II (cm^-1^) | Inferred secondary structure | Spectral interpretation | References |
| --- | --- | --- | --- | --- | --- |
| Control | 1666 | 1527 | β-turn/β-sheet components | Native protein conformation with balanced α/β and turn structures typical of stromal collagen | Barth & Zscherp 2002, *Q Rev Biophys*, 35(4):369–430. [10.1017/S0033583502003815](https://doi.org/10.1017/S0033583502003815) |
| 3 mA | 1674 | 1535 | Turn-dominated | Slight blue shift indicates tighter hydrogen bonding and increased β-turn or loop ordering | Jackson & Mantsch 1995, *Crit Rev Biochem Mol Biol*, 30(2):95–120. [10.3109/10409239509085142](https://doi.org/10.3109/10409239509085142) |
| 4 mA | 1682 | 1551 | β-turns + β-sheet contributions | Further blue shift; possible aggregation or increased inter-strand H-bonding characteristic of β-sheets | Surewicz & Mantsch 1988, *Biochim Biophys Acta*, 952(2):115–130. [10.1016/0167-4838(88)90107-2](https://doi.org/10.1016/0167-4838(88)90107-2) |
| 5 mA | 1666 | 1527 | Mixed α/β equilibrium (native-like) | Frequencies revert to control values, indicating partial structural recovery or adaptive refolding | Arrondo et al. 1993, *Prog Biophys Mol Biol*, 59(1):23–56. [10.1016/0079-6107(93)90006-6](https://doi.org/10.1016/0079-6107(93)90006-6) |
| 6 mA | 1659 | 1527 | α-helix-enriched | Downshift of amide I consistent with α-helical content or weakened inter-strand H-bonds; moderate unfolding | Kong & Yu 2007, *Acta Biochim Biophys Sin*, 39(8):549–559. [10.1111/j.1745-7270.2007.00320.x](https://doi.org/10.1111/j.1745-7270.2007.00320.x) |
| 7 mA | 1659 | 1512 | Disordered / unfolded | Simultaneous red shift of both bands indicates disruption of H-bonding, protein unfolding, or loss of tertiary order | Jackson & Mantsch 1995, *Crit Rev Biochem Mol Biol*, 30(2):95–120. [10.3109/10409239509085142](https://doi.org/10.3109/10409239509085142) |
| 500 mA | 1674 |  | Denatured / aggregated | Loss of amide II and blue-shifted amide I imply irreversible denaturation, dehydration, or covalent modification | Fabian et al. 1999, *Biopolymers*, 50(5):461–476. [10.1002/(SICI)1097-0282(19991005)50:5<461::AID-BIP4>3.0.CO;2-5](https://doi.org/10.1002/(SICI)1097-0282(19991005)50:5%3C461::AID-BIP4%3E3.0.CO;2-5) |
