# Supplementary material for: Ex vivo and computational investigation of corneal iontophoresis to enhance penetration of high-molecular-weight compounds: a study using albumin as a model molecule
Source: Sci Rep. 2026 Mar 31;16:10990. doi: 10.1038/s41598-026-43580-y (PMC13043707; doi:10.1038/s41598-026-43580-y)
Supplement: Supplementary file 3 — Supplementary Material 3 [file 41598_2026_43580_MOESM3_ESM.docx]

Table 4: Integrated area percentage calculated from the FTIR-second derivative spectra after transcorneal iontophoresis

|  | _str_OH | _asy_OH | _sy_OH | Total OH |
| --- | --- | --- | --- | --- |
| Control | 32.14 ± 0.9 | 4.7 ± 0.7 | 2.2 ± 0.5 | 36.96 ± 3.3 |
| 0.5 mA | 34.9 ± 3.1 | 5.1 ± 0.3 | 2.4 ± 0.2 | 42.4 ± 3.9 |
| 1 mA | 33.6 ± 2.6 | 5.9 ± 0.9 | 2.25 ± 0.12 | 41.75 ± 3.7 |
| 2 mA | 33.5 ± 1.9 | 4.94 ± 0.7 | 2.4 ± 0.4 | 40.84 ± 4.2 |
| 3 mA | ^†^26.8 ± 1.1 | ^†^0.4 ± 0.003 | ^†^0.6 ± 0.007 | ^†^26.8 ± 2.5 |
| 4 mA | ^†^18.7 ± 1.4 | 6.6 ± 0.8 | 2.3 ± 0.6 | ^†^27.6 ± 2.6 |
| 5 mA | ^†^14.9 ± 2.1 | 4.3 ± 0.3 | ^†^0.2 ± 0.001 | ^†^19.2 ± 2.4 |
| 6 mA | ^†^27 ± 1.6 | ^†^0.31 ± 0.002 | 2 ± 0.7 | ^†^31.31 ± 1.9 |
| 7 mA | ^†^18.2 ± 1.6 | ^†^0.5 ± 0.003 | ^†^8.3 ± 0.3 | ^†^26.5 ± 2.2 |
| 500 mA | ^†^18.4 ± 2.2 | 5.8 ± 0.6 | ^†^4.2 ± 0.14 | ^†^28.4 ± 2.2 |

^†^ Statistically significant relative to the control
